# Lifestyle Factors Associated with Children’s and Adolescents’ Adherence to the Mediterranean Diet Living in Mediterranean Countries: The DELICIOUS Project

**DOI:** 10.3390/nu17010026

**Published:** 2024-12-25

**Authors:** Alice Rosi, Francesca Scazzina, Francesca Giampieri, Ludwig Álvarez-Córdova, Osama Abdelkarim, Achraf Ammar, Mohamed Aly, Evelyn Frias-Toral, Juancho Pons, Laura Vázquez-Araújo, Carmen Lili Rodríguez Velasco, Julién Brito Ballester, Lorenzo Monasta, Ana Mata, Adrián Chacón, Pablo Busó, Giuseppe Grosso

**Affiliations:** 1 Human Nutrition Unit, Department of Food and Drug, University of Parma, 43124 Parma, Italy; 2 Department of Clinical Sciences, Università Politecnica delle Marche, 60131 Ancona, Italy; 3 Research Group on Food, Nutritional Biochemistry and Health, Universidad Europea del Atlántico, Isabel Torres 21, 39011 Santander, Spain; 4 Joint Laboratory on Food Science, Nutrition, and Intelligent Processing of Foods, Polytechnic University of Marche, Universidad Europea del Atlántico Spain and Jiangsu University, 60121 Ancona, Italy; 5 International Research Center for Food Nutrition and Safety, Jiangsu University, Zhenijang 212013, China; 6 Carrera de Nutrición y Dietética, Facultad de Ciencias de la Salud, Universidad Católica de Santiago de Guayaquil, Av. Pdte. Carlos Julio Arosemena Tola, Guayaquil 090615, Ecuador; 7 Facultad de Ciencias de la Salud, Universidad de Las Américas (UDLA), Quito 170513, Ecuador; 8 Faculty of Physical Education, Assiut University, Assiut 71515, Egypt; 9 Department of Training and Movement Science, Institute of Sport Science, Johannes Gutenberg-University Mainz, 55122 Mainz, Germany; 10 Research Laboratory, Molecular Bases of Human Pathology, LR19ES13, Faculty of Medicine, University of Sfax, Sfax 3029, Tunisia; 11 School of Medicine, Universidad Espíritu Santo, Samborondón 091952, Ecuador; 12 Editorial Luis Vives (EDELVIVES), Carretera de Madrid, 50012 Zaragoza, Spain; 13 BCC Innovation, Technology Center in Gastronomy, Basque Culinary Center, 20009 Donostia-San Sebastián, Spain; 14 Basque Culinary Center, Faculty of Gastronomic Sciences, Mondragon Unibertsitatea, 20009 Donostia-San Sebastián, Spain; 15 Department of Health, Nutrition and Sport, Universidad Internacional Iberoamericana, Campeche 24560, Mexico; 16 Department of Project Management, Universidade Internacional do Cuanza, Cuito EN 250, Bié, Angola; 17 Faculty of Social Sciences and Humanities, Universidad Internacional Iberoamericana, Arecibo, PR 00613, USA; 18 Faculty of Health Sciences, Universidad de La Romana, La Romana 22000, Dominican Republic; 19 Institute for Maternal and Child Health—IRCCS Burlo Garofolo, 34137 Trieste, Italy; 20 Technological Institute for Children’s Products & Leisure AIJU, 03440 Alicante, Spain; 21 Department of Biomedical and Biotechnological Sciences, University of Catania, 95123 Catania, Italy; 22 Center for Human Nutrition and Mediterranean Foods (NUTREA), University of Catania, 95123 Catania, Italy

**Keywords:** Mediterranean diet, determinants, children

## Abstract

**Background/Objectives.** Traditional dietary patterns are being abandoned in Mediterranean countries, especially among younger generations. This study aimed to investigate the potential lifestyle determinants that can increase adherence to the Mediterranean diet in children and adolescents. **Methods.** This study is a cross-sectional analysis of data from five Mediterranean countries (Italy, Spain, Portugal, Egypt, and Lebanon) within the context of the EU-funded project DELICIOUS (UnDErstanding consumer food choices & promotion of healthy and sustainable Mediterranean Diet and LIfestyle in Children and adolescents through behavIOUral change actionS). This study comprised information on 2011 children and adolescents aged 6–17 years old collected during 2023. The main background characteristics of both children and parents, including age, sex, education, and family situation, were collected. Children’s eating (i.e., breakfast, place of eating, etc.) and lifestyle habits (i.e., physical activity level, sleep, and screen time) were also investigated. The level of adherence to the Mediterranean diet was assessed using the KIDMED index. Logistic regression analyses were performed to test for likelihood of higher adherence to the Mediterranean diet. **Results.** Major determinants of higher adherence to the Mediterranean diet were younger age, higher physical activity level, adequate sleep duration, and, among dietary habits, having breakfast and eating with family members and at school. Parents’ younger age and higher education were also determinants of higher adherence. Multivariate adjusted analyses showed that an overall healthier lifestyle and parents’ education were the factors independently associated with higher adherence to the Mediterranean diet. **Conclusions.** Higher adherence to the Mediterranean diet in children and adolescents living in the Mediterranean area is part of an overall healthy lifestyle possibly depending on parents’ cultural background.

## 1. Introduction

The Mediterranean dietary pattern refers to the dietary habits evolved over centuries adopted by the individuals living in the Mediterranean area [1]. Although there is no univocal definition due to the diversity in cultural and heritage background, some common characteristics include high consumption of fruits, vegetables, legumes, nuts, and whole grains; low consumption of red meat and sweets; extra virgin olive oil as the main source of fat; and moderate consumption of dairy products, poultry, eggs, and fish as sources of animal proteins [2]. The Mediterranean diet is also an example of a sustainable diet in which seasonal and traditional foods and biodiversity are favored [3,4]. Scientific evidence has demonstrated that higher adherence to the Mediterranean diet is associated with a lower risk of noncommunicable diseases, including cardiovascular disease [5], metabolic syndrome and its components [6,7], neurodegenerative diseases [8], and preventive effects against certain cancers [9]; overall, the adoption of this dietary pattern has been related to a prolonged lifespan [10]. The mechanisms related to the benefits of the Mediterranean diet rely on an optimal balance between nutrients, including healthy unsaturated fats, fiber, and limitation of trans fats and refined sugars, as well as richness in plant-based products, providing a large variety of vitamins and potentially beneficial bioactive compounds [11,12]. Concerning children and adolescents, a recent meta-analysis of randomized clinical trials investigating the effect of Mediterranean-diet-based interventions has confirmed its efficiency in reducing body mass index and obesity [13], resulting in higher quality of life [14]. The benefits associated with the Mediterranean diet could be related to the anti-inflammatory and antioxidant properties of the many components of such plant-based dietary patterns, as well as a proper balance between macro- and micronutrients, fiber content, and limited contribution to energy intake of unhealthy fats and sugars [15]. Notwithstanding the aforementioned benefits, while diet quality has been registered as being improved globally [16], adherence to the Mediterranean diet has decreased over time [17] and has been shown to suffer from poor popularity among children and adolescents living in Mediterranean countries [18]. Aside from diet quality, concerns regarding engagement in overall unhealthy lifestyles have been raised over the last decades [19]. Regarding eating habits, it has been observed that skipping breakfast [20] and out-of-home eating [21] are associated with higher prevalence of obesity and increased risk of becoming overweight or obese, respectively. Similarly, the frequency of school and family meals is associated with better diet quality and may reduce the risk of obesity and poorer mental health and wellbeing [22]. On top of that, sedentary behaviors (such as screen time) and lack of physical activity have been reported worldwide, with about half the after-school time spent in sedentary activities [23]. Curiously, higher rates of scarce physical activity have been reported in Mediterranean countries, where the climate and the traditional lifestyle could actually promote opposite behaviors [24]. Also, unhealthy lifestyles established during childhood have been reported to form the foundation for such behaviors in the future [25]. Overall, intervening at an early stage in order to establish healthy eating behaviors during childhood, has been considered a good strategy to prevent age-related noncommunicable diseases in adulthood [26]. The aim of this study was to identify the main factors that can influence the adherence to the Mediterranean diet in children and adolescents in five Mediterranean countries.

## 2. Materials and Methods

### 2.1. Study Design and Population

This cross-sectional analysis was carried out in the framework of the European-funded DELICIOUS (UnDErstanding consumer food choices & promotion of healthy and sustainable Mediterranean Diet and LIfestyle in Children and adolescents through behavIOUral change actionS) project [27]. For the purposes of this study, a preliminary survey was conducted among parents of 6–17-year-old children and adolescents from five Mediterranean countries (Italy, Spain, Portugal, Egypt, and Lebanon). Participants were enrolled on a voluntary basis from the network of collaborators of the Technological Institute for Children’s Products & Leisure (AIJU). Inclusion criteria were the following: (i) having children fitting the target population in terms of age range and (ii) having access to the internet to fill out the survey. There were no exclusion criteria once the volunteers agreed to be enrolled in the survey. Taking into account similar studies conducted in Mediterranean countries with similar goals [28,29,30,31,32], a target of about 400 individuals per each Mediterranean country was estimated to be sufficient to detect significant differences across groups of exposed individuals. However, given the variety of variables investigated and the voluntary nature of the participation in the survey, the sample cannot be considered truly representative of a random population but, rather, indicative of the target one. Data were collected via an electronic survey filled out by the parents, which provided an informed consent prior to enrollment in the survey. All procedures were carried out according to the Declaration of Helsinki (1989) of the World Medical Association.

### 2.2. Data Collection

Each participant was asked to report data regarding demographic and lifestyle characteristics: parents’ sex, age, education, and occupation and children’s sex, age, anthropometric measures, eating habits, level of physical activity, and screen and sleep time. Parents’ education was categorized as (i) low (primary), (ii) medium (secondary), (iii) and high (tertiary), while occupation was categorized as (i) unemployed and (ii) currently working. Based on the guidelines of the Centers for Disease Control and Prevention (CDC) weight-for-height growth charts percentiles for children and teens ages 2 through 19 years [33], sex- and age-related body mass index (BMI) of children/adolescents was calculated from obtained height and weight, and it was used to categorize children/adolescents as (i) normal weight (BMI 5th-84th percentile), (ii) overweight (BMI 85th-94th percentile), and (iii) obese (BMI ≥ 95th percentile for children and teens of the same age and sex). Lifestyle quality of children/adolescents was assessed using the Electronic Kids Dietary Index (E-KINDEX), which incorporates 3 main domains of questions about (i) food groups intake (13 items), (ii) eating beliefs and behaviors (8 items), and (iii) dietary practices (9 items) [34]. For the purposes of this study, only domains related to lifestyle were used (as food groups intake represented the outcome of interest). Physical activity level was assessed using The International Physical Activity Questionnaire-Short Form (IPAQs), referring to the last 7 days before compilation about three specific types of activities (walking, moderate-intensity activities, and vigorous-intensity activities), with frequency (measured in days per week) and duration (time per day) collected separately for each activity type [35]. Finally, based on the recommendations of the National Sleep Foundation concerning the optimum sleeping time in children and teenagers, sleep duration (in hours) has been categorized as follows: (i) <8 h, (ii) 8–10 h, and (iii) >10 h [36], while screen time (in hours) was categorized as (i) <2 h/day, (ii) 2–4 h/day, and (iii) >4 h/day.

### 2.3. Dietary Assessment and Mediterranean Diet Adherence

Using a semi-structured 24 h recall, dietary information was collected. Participants were asked the type of foods and drinks for each meal (including snacks) their offspring had consumed in the previous 24 h among 20 main food groups provided (fruit, fresh and cooked vegetables, olive oil, pasta or rice, red meat, white meat, tea, coffee, chocolate, juices, legumes, fish and seafoods, cakes and biscuits/pastries, milk, yogurt, nuts, whole grains, eggs, junk foods, and fast foods) and an open-ended space for additional foods. Consumption of foods occurring over the time span of a week was assessed through food frequency questions including the most common food items. Adherence to the Mediterranean diet was assessed through the Mediterranean Diet Quality Index (KIDMED) test, which is a 16 yes/no question assay on dietary habits. Answers with questions that had a negative connotation towards the Mediterranean diet received a score of −1, while those with questions that indicated a Mediterranean habit received a score of +1, reaching a possible total score of 12. According to the literature [37], the KIDMED score was divided into categories and a score ≥7 was deemed as having high adherence to the Mediterranean diet.

### 2.4. Statistical Analysis

Categorical variables were provided as absolute numbers and relative frequencies, with differences between background characteristics tested through the chi-squared test. Logistic regression analyses were performed to test the association between the variables of interest and high adherence to the Mediterranean diet by calculating the odds ratios (ORs) and 95% confidence intervals (CIs). Variables of interest were grouped and adjusted based on macro domains (i.e., demographic characteristics of parents and children/adolescents, eating behaviors, and lifestyle habits). An additional multivariate analysis was conducted with those variables that showed significance in each domain. All *p*-values were reported as two-sided and considered significant when <0.05. SPSS 28 (SPSS Inc., Chicago, IL, USA) software was used for all the statistical tests.

## 3. Results

A total of 2011 participants were recruited. Based on parents’ responses, 865 children and adolescents had been reported to have high adherence to the Mediterranean diet. The main demographic characteristics of the study population (parents and children/adolescents) according to adherence to the Mediterranean diet are presented in Table 1. A higher proportion of younger children aged between 6 and 8 years (*p* = 0.017), female sex respondents (*p* = 0.006), and those with a higher parental educational level (*p* < 0.001) and older age (*p* < 0.001) were found among those with higher adherence to the Mediterranean diet (Table 1). A multivariate analysis of these variables showed that those whose parents had a higher level of education were significantly associated with high adherence to the Mediterranean diet (OR = 2.11, 95% CI: 1.15, 3.88). However, a significant negative association with high Mediterranean diet adherence was also found in participants aged between 12 and 14 years (OR = 0.72, 95% CI: 0.54, 0.98), those that were obese (OR = 0.68, 95% CI: 0.50, 0.93), and those with older parents (OR = 0.64, 95% CI: 0.48, 0.85).

The eating habits of participants according to level of adherence to the Mediterranean diet and their associations are presented in Table 2. There was a significant difference in adherence to the Mediterranean diet, with higher rates among children and adolescents always having breakfast (*p* < 0.001) and eating more frequently with family members (*p* = 0.004) and at school (*p* = 0.010). Regarding the association between the variables, having breakfast (OR = 3.03, 95% CI: 2.18, 4.21), out-of-home eating (OR = 1.66, 95% CI: 1.18, 2.33), and eating with family members (OR = 2.49, 95% CI: 1.10, 5.63) were associated with higher Mediterranean diet adherence.

The lifestyle habits of children/adolescents according to Mediterranean diet adherence and the association between these potential determinants and high adherence to the Mediterranean diet are presented in Table 3. A higher proportion of individuals with optimal sleep duration, between 8 and 10 h (*p* < 0.001), had higher Mediterranean diet adherence; however, in children and adolescents with lower physical activity levels (*p* < 0.001) and lower healthy lifestyle score (based on the E-KINDEX score, *p* = 0.021), a lower adherence to the Mediterranean diet was reported. A significant association between higher sleep duration (OR = 1.61, 95% CI: 1.01, 2.56), intermediate level of physical activity (OR = 2.24, 95% CI: 1.78, 2.21), higher healthy lifestyle score (OR = 1.28, 95% CI: 1.03, 1.60), and higher adherence to the Mediterranean diet was registered.

**Figure 1:**
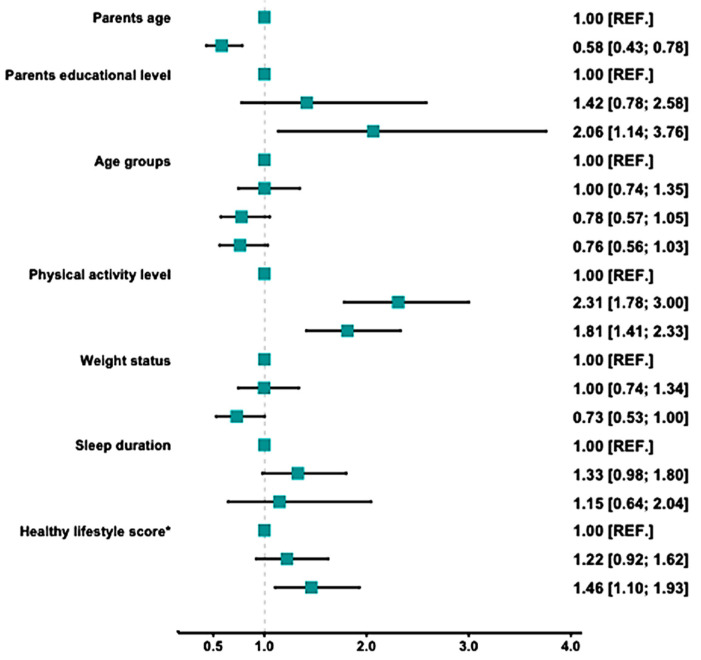
Multivariate analysis of factors associated with high adherence to the Mediterranean diet. * Healthy lifestyle score based on the E-KINDEX score.

Figure 1 shows the multivariate analysis of the major determinants of higher adherence to the Mediterranean diet. A significant association between higher parental education (OR = 2.06, 95% CI: 1.14, 3.76), intermediate physical activity level (OR = 2.31, 95% CI: 1.78, 3.00), and higher healthy lifestyle scores (OR = 1.46, 95% CI: 1.10, 1.93) was confirmed.

No significant differences in the association between the potential Mediterranean diet determinants (i.e., parents’ age, parents’ educational level, age groups, physical activity level, sleep duration, and healthy lifestyle score) and high adherence to the Mediterranean diet were found among the countries involved in this study (Supplementary Table S1).

## 4. Discussion

The present study investigated factors associated with adherence to the Mediterranean diet of children and adolescents living in five Mediterranean countries. The aim of this study was to highlight key findings concerning demographic and other potential influencing factors in order to identify potential targets for strategies for improvement. Adherence to the Mediterranean diet in the study population has been recently reported to be lower than one could expect in Mediterranean countries and similar across countries included in this study [38]. Among major food groups poorly consumed, fruit, vegetables, legumes, and cereals have been reported to be generally underrepresented in the diets of Mediterranean children and adolescents [39]. Recent studies reported slightly worse estimates, documenting a noticeable decline in adherence to the Mediterranean diet in Mediterranean countries, with high adherence ranging from 20% to 40% of children and adolescents in the countries involved in the present study [40,41,42,43]. Notably, the level of adherence to the Mediterranean diet in Mediterranean countries also suffered from the changes in lifestyle due to the recent COVID-19 pandemic (disrupted daily routines, including meal patterns, physical activity, and school attendance), leading to an overall worsening of dietary habits, especially among younger age groups [44]. The results showed that higher adherence to the Mediterranean diet is rooted within an overall healthier lifestyle, including adequate physical activity, sufficient sleep and reduced screen time, and other specific eating habits, such as having breakfast and eating with family and at school. These findings are in line with the scientific literature published so far reporting that adherence to the Mediterranean diet has been generally influenced by both eating and lifestyle habits [45,46,47,48].

Among eating habits, having breakfast has long been hypothesized as a potential factor associated with adherence to the Mediterranean diet [49]. A multinational cross-sectional study involving different European countries showed that breakfast consumers had higher adherence to the Mediterranean diet than breakfast-skippers [50]. Similar results were found in a Lebanese cross-sectional study where breakfast-skippers had lower adherence to the Mediterranean diet [51]. However, recently, other aspects more related to the psychosocial domains of eating habits have gained interest: eating habits promoting conviviality, such as meals with parents or at school, have in fact been suggested to be related to higher adherence to the Mediterranean diet [52].

Among lifestyle factors, level of physical activity is among the most studied among younger generations for its association with dietary habits and a target for interventions to improve children’s and adolescents’ health [53]. Having an active lifestyle in childhood is important to estimate the risk of obesity in the upcoming puberty, a vulnerable period due to hormonal and lifestyle changes [54]. In fact, sedentary behaviors have been associated with unfavorable body composition and higher clustered cardiometabolic risk factors [55]. By contrast, a recent meta-analysis on children and adolescents showed that physical activity was associated with higher adherence to the Mediterranean diet and better general health [56]. With specific reference to those countries investigated in this study, a recent survey conducted in Lebanon regarding the role of some determinants on Mediterranean diet adherence underlined that a higher physical activity level was associated with greater adherence [57]. Similarly, several reports from Spain showed that individuals with higher physical activity levels were more likely to adopt a Mediterranean diet [58,59,60]. These trends have also previously been reported in Italian and Portuguese children and adolescents [49,61,62,63,64,65]. Also, the importance of having correct sleep hygiene [62,63,64] and reduced screen-time-related sedentary behaviors [66] is hypothesized to potentially be associated with higher adherence to the Mediterranean diet and prevention of obesity. Some studies specifically conducted on Spanish [58,67,68,69,70], Italian [31,71], Portuguese [43], and Lebanese [43] children and adolescents just support the hypothesis of an association between sleep quality (including sleeping time and length), reduced screen time, and higher adherence to the Mediterranean diet.

While most evidence suggests an association between lifestyle habits and adherence to the Mediterranean diet, we hypothesized that other background characteristics may be involved in determining dietary habits. In fact, our findings showed that family background characteristics may play a determinant role in such matters. A large number of studies suggest that adherence to the Mediterranean diet is consistently related to the demographic and social characteristics of both children and parents [68,72,73]. Some reviews have been performed regarding social factors affecting adherence to a healthy eating pattern in young adults [74,75] and eating behavior in children, emphasizing how different social factors, such as family environment, parental influences, education, and economic status, could affect children’s food choices [76]. A large share of the literature agrees that parent educational level represents a crucial factor associated with higher adherence to the Mediterranean diet [77]. Concerning the countries directly involved in this study, previous cross-sectional analyses from primary school children with the purpose of investigating the association between parents’ lifestyle determinants and children’s dietary habits reported that higher education of the mothers was an important determinant of their children’s adherence to the Mediterranean diet [29,78,79]. Similar determinants of adherence to the Mediterranean diet have also been reported in Spanish children [67] and adolescents [80]. Interestingly, a comparative analysis of two cross-sectional nationwide representative studies investigating how children’s and adolescents’ dietary patterns have changed over 20 years in Spain reported that diet quality has worsened especially when parents’ education was not university level [81]. Based on the data available in the scientific literature, it can be hypothesized that parents with a higher level of education could have more nutritional knowledge and positively influence adherence to the Mediterranean diet in their children [82]. In addition, parents with higher educational levels could have more motivation toward healthier choices to pass on to their offspring. Several studies in fact have underlined that adherence to a Mediterranean dietary pattern increased with greater nutrition knowledge [83,84,85].

To effectively promote adherence to the Mediterranean diet (MD) among children, interventions should be designed to address key factors such as parental education, sleep duration, and overall lifestyle behaviors. As evidence suggests that high parental education and active engagement in health-promoting behaviors are strong predictors of children’s dietary habits, public health strategies should prioritize parental education programs that emphasize the importance of a balanced diet, particularly the Mediterranean dietary pattern. These programs can be integrated into community health initiatives, schools, and primary care settings. For example, providing parents with resources such as workshops, printed materials, and digital platforms focused on meal planning, cooking skills, and the nutritional benefits of traditional Mediterranean foods could foster positive dietary habits in children. Additionally, strategies aimed at enhancing the nutritional literacy of parents may contribute to the creation of a supportive home environment, which is crucial in shaping children’s eating behaviors. Promoting parental role models who consistently follow the Mediterranean diet could also reinforce these healthy eating patterns within families. In addition, the content of such interventions should include the promotion of healthy lifestyles in general, which encompasses factors like sleep hygiene, physical activity, limited screen time, and balanced eating. For instance, schools could increase opportunities for physical activity through daily recess, sports programs, or after-school physical activity clubs, while also limiting the availability of unhealthy foods in school cafeterias. Public health campaigns could also promote the benefits of limiting screen time and engaging in active play, highlighting how these behaviors positively influence both sleep and dietary habits.

The current study presents strengths and limitations that should be considered. The main strength is represented by the multinational sample retrieved around the geographical Mediterranean area. Nevertheless, the cross-sectional analysis only permits estimating the association between background characteristics, eating and lifestyle habits, and Mediterranean diet adherence but not the casualty relation. Moreover, data collection through questionnaires administered to the parents referring to their children may be subjected to memory and social desirability bias. Additionally, data are self-reported; thus, it may also lack accuracy (i.e., concerning measures). Finally, given the voluntary participation and the large number of variables investigated, we cannot establish whether the sample collected is truly representative of the general population it refers to.

## 5. Conclusions

In conclusion, exploring current eating and lifestyle habits in children and adolescents might be useful to explore determinants of adherence to the Mediterranean diet and, more generally, engagement in healthy or unhealthy lifestyle behaviors. Identifying potential determinants of adherence to healthy dietary patterns may represent a valid aid to select important targets of multidisciplinary interventions in children and adolescents. Efforts to improve adherence to the Mediterranean diet in European youth could benefit from targeted interventions that address the specific determinants identified in these studies. Promoting physical activity, reducing screen time, and enhancing nutrition education both at home and in schools are crucial steps. Incorporating these strategies into a comprehensive intervention framework such as the DELICIOUS project itself could enhance Mediterranean diet adherence in children, not only by targeting dietary behaviors directly but also by addressing the broader environmental and lifestyle factors that influence children’s health. These efforts should be designed to be accessible, sustainable, and adaptable across various socio-economic and cultural contexts to ensure their widespread impact. Additionally, policies aimed at making healthy, Mediterranean-diet-compatible foods more accessible and affordable can help foster better adherence. Growing interest from governmental bodies is warranted to tackle the burden of children’s and adolescents’ malnutrition and adoption of behaviors related to detrimental effects in the future.

## Figures and Tables

**Table 1:** Demographic characteristics of parents and children/adolescents participating in the study by level of adherence to the Mediterranean diet (n = 2011).

	Adherence to Mediterranean Diet			High Adherence to Mediterranean Diet
	Low	High	*p-Value*	OR (95% CI) *
Age groups, (n, %)			*0.017*	
6–8 y	296 (25.8)	249 (28.8)		1
9–11 y	267 (23.3)	235 (27.2)		1.00 (0.75, 1.35)
12–14 y	282 (24.6)	196 (22.7)		0.72 (0.54, 0.98)
15–17 y	301 (26.3)	185 (21.4)		0.73 (0.54, 0.99)
Sex, (n, %)			*0.472*	
Male	575 (50.2)	420 (48.6)		1
Female	571 (49.8)	445 (51.4)		0.96 (0.78, 1.19)
Weight status, (n, %)			*0.159*	
Normal weight	599 (68.6)	488 (68.8)		1
Overweight	135 (15.5)	128 (18.1)		1.02 (0.76, 1.36)
Obese	139 (15.9)	93 (13.1)		0.68 (0.50, 0.93)
Parent’s age			*<0.001*	
<44 y	204 (17.8)	219 (25.3)		1
≥45 y	942 (82.2)	646 (74.7)		0.64 (0.48, 0.85)
Parents occupation, (n, %)			*0.060*	
Unemployed	854 (75.8)	679 (79.4)		1
Current working	272 (24.2)	176 (20.6)		0.91 (0.69, 1.20)
Parents education, (n, %)			*<0.001*	
Low	68 (6.2)	23 (2.7)		1
Medium	467 (42.6)	283 (33.8)		1.48 (0.80, 2.72)
High	561 (51.2)	532 (63.5)		2.11 (1.15, 3.88)

* Analyses were adjusted for all variables presented in the table.

**Table 2:** Eating behaviors of children/adolescents by level of adherence to the Mediterranean diet (n = 2011).

	Adherence to Mediterranean Diet			High Adherence to Mediterranean Diet
	Low	High	*p-Value*	OR (95% CI) *
Breakfast habit, (n, %)			*<0.001*	
Never/seldom	209 (18.2)	68 (7.9)		1
Often	204 (17.8)	144 (16.6)		2.06 (1.45, 2.92)
Always	733 (64.0)	653 (75.5)		3.03 (2.18, 4.21)
Eating with family, (n, %)			*0.004*	
Seldom	33 (2.9)	8 (0.9)		1
Often	353 (30.8)	248 (28.7)		2.51 (1.11, 5.70)
Daily	760 (66.3)	609 (70.4)		2.49 (1.10, 5.63)
Eating alone, (n, %)			*0.842*	
Never/seldom	710 (62.0)	537 (62.1)		1
Often	344 (30.0)	253 (29.2)		1.20 (0.92, 1.56)
Daily	92 (8.0)	75 (8.7)		1.12 (0.79, 1.59)
Eating at school, (n, %)			*0.010*	
Never/seldom	504 (44.0)	327 (37.8)		1
Often	349 (30.5)	273 (31.6)		1.23 (0.98, 1.55)
Almost daily	293 (25.6)	265 (30.6)		1.12 (0.79, 1.59)
Eating advertised foods, (n, %)			*0.261*	
No	600 (52.4)	431 (49.8)		1
Yes	546 (47.6)	434 (50.2)		1.14 (0.93, 1.40)
Eating home-made foods, (n, %)			*0.057*	
Seldom	159 (13.9)	93 (10.8)		1
Often	512 (44.7)	379 (43.8)		1.19 (0.87, 1.62)
Almost daily	475 (41.4)	393 (45.4)		1.25 (0.93, 1.68)

*Analyses were adjusted for all variables presented in the table.

**Table 3:** Lifestyle behaviors of children/adolescents by level of adherence to the Mediterranean diet (n = 2011).

	Adherence to Mediterranean Diet			High Adherence to Mediterranean Diet
	Low	High	*p-Value*	OR (95% CI) **
Sleep duration, n (%)			*<0.001*	
Less than 8 h	246 (21.5)	125 (14.5)		1
8–10 h	846 (73.8)	696 (80.5)		1.46 (1.14, 1.86)
>10 h	54 (4.7)	44 (5.1)		1.61 (1.01, 2.56)
Screen time (n, %)			*0.211*	
<2 h/day	650 (56.7)	481 (55.6)		1
2–4 h/day	398 (34.7)	325 (37.6)		1.06 (0.88, 1.29)
>4 h/day	98 (8.6)	59 (6.8)		0.85 (0.60, 1.22)
Physical activity level, (n, %)			*<0.001*	
Low	666 (58.1)	351 (40.6)		1
Medium	209 (18.2)	252 (29.1)		2.24 (1.78, 2.81)
High	271 (23.6)	262 (30.3)		1.78 (1.43, 2.21)
Healthy lifestyle score *, (n, %)			*0.021*	
Low	467 (40.8)	311 (36.0)		1
Medium	332 (29.0)	244 (28.2)		1.16 (0.93, 1.46)
High	347 (30.3)	310 (35.8)		1.28 (1.03, 1.60)

* based on the E-KINDEX score. **Analyses were adjusted for all variables presented in the table.

## Data Availability

The data presented in this study are available on request from the corresponding author upon reasonable request due to data confidentiality.

## Supplementary Materials

The following supporting information can be downloaded at: https://www.mdpi.com/article/10.3390/nu17010026/s1, Table S1: Factors associated with high adherence to the Mediterranean diet in individual countries participating in the study.

## References

[1] Godos J., Scazzina F., Paternò Castello C., Giampieri F., Quiles J.L., Briones Urbano M., Battino M., Galvano F., Iacoviello L., de Gaetano G. (2024). Underrated aspects of a true Mediterranean diet: Understanding traditional features for worldwide application of a “Planeterranean” diet. J. Transl. Med..

[2] Bach-Faig A., Berry E.M., Lairon D., Reguant J., Trichopoulou A., Dernini S., Medina F.X., Battino M., Belahsen R., Miranda G. (2011). Mediterranean Diet Foundation Expert Group Mediterranean diet pyramid today. Science and cultural updates. Public Health Nutr..

[3] Dernini S., Berry E.M., Serra-Majem L., La Vecchia C., Capone R., Medina F.X., Aranceta-Bartrina J., Belahsen R., Burlingame B., Calabrese G. (2017). Med Diet 4.0: The Mediterranean diet with four sustainable benefits. Public Health Nutr..

[4] Tolomeo M., De Carli L., Guidi S., Zanardi M., Giacomini D., Devecchi C., Pistone E., Ponta M., Simonetti P., Sykes K. (2023). The Mediterranean Diet: From the pyramid to the circular model. Med. J. Nutr. Metab..

[5] Grosso G., Marventano S., Yang J., Micek A., Pajak A., Scalfi L., Galvano F., Kales S.N. (2017). A comprehensive meta-analysis on evidence of Mediterranean diet and cardiovascular disease: Are individual components equal?. Crit. Rev. Food Sci. Nutr..

[6] Godos J., Zappalà G., Bernardini S., Giambini I., Bes-Rastrollo M., Martinez-Gonzalez M. (2017). Adherence to the Mediterranean diet is inversely associated with metabolic syndrome occurrence: A meta-analysis of observational studies. Int. J. Food Sci. Nutr..

[7] Papadaki A., Nolen-Doerr E., Mantzoros C.S. (2020). The Effect of the Mediterranean Diet on Metabolic Health: A Systematic Review and Meta-Analysis of Controlled Trials in Adults. Nutrients.

[8] Huang L., Tao Y., Chen H., Chen X., Shen J., Zhao C., Xu X., He M., Zhu D., Zhang R. (2023). Mediterranean-Dietary Approaches to Stop Hypertension Intervention for Neurodegenerative Delay (MIND) Diet and Cognitive Function and its Decline: A Prospective Study and Meta-analysis of Cohort Studies. Am. J. Clin. Nutr..

[9] Schwingshackl L., Schwedhelm C., Galbete C., Hoffmann G. (2017). Adherence to Mediterranean Diet and Risk of Cancer: An Updated Systematic Review and Meta-Analysis. Nutrients.

[10] Dinu M., Pagliai G., Casini A., Sofi F. (2018). Mediterranean diet and multiple health outcomes: An umbrella review of meta-analyses of observational studies and randomised trials. Eur. J. Clin. Nutr..

[11] Grosso G., Laudisio D., Frias-Toral E., Barrea L., Muscogiuri G., Savastano S., Colao A. (2022). Anti-Inflammatory Nutrients and Obesity-Associated Metabolic-Inflammation: State of the Art and Future Direction. Nutrients.

[12] Farias-Pereira R., Zuk J.B., Khavaran H. (2023). Plant bioactive compounds from Mediterranean diet improve risk factors for metabolic syndrome. Int. J. Food Sci. Nutr..

[13] López-Gil J.F., García-Hermoso A., Sotos-Prieto M., Cavero-Redondo I., Martínez-Vizcaíno V., Kales S.N. (2023). Mediterranean Diet-Based Interventions to Improve Anthropometric and Obesity Indicators in Children and Adolescents: A Systematic Review with Meta-Analysis of Randomized Controlled Trials. Adv. Nutr..

[14] Romero-Robles M.A., Ccami-Bernal F., Ortiz-Benique Z.N., Pinto-Ruiz D.F., Benites-Zapata V.A., Casas Patiño D. (2022). Adherence to Mediterranean diet associated with health-related quality of life in children and adolescents: A systematic review. BMC Nutr..

[15] Tosti V., Bertozzi B., Fontana L. (2018). Health benefits of the mediterranean diet: Metabolic and molecular mechanisms. J. Gerontol. A Biol. Sci. Med. Sci..

[16] Miller V., Webb P., Cudhea F., Shi P., Zhang J., Reedy J., Erndt-Marino J., Coates J., Mozaffarian D. (2022). Global Dietary Database Global dietary quality in 185 countries from 1990 to 2018 show wide differences by nation, age, education, and urbanicity. Nat. Food.

[17] Damigou E., Faka A., Kouvari M., Anastasiou C., Kosti R.I., Chalkias C., Panagiotakos D. (2023). Adherence to a Mediterranean type of diet in the world: A geographical analysis based on a systematic review of 57 studies with 1,125,560 participants. Int. J. Food Sci. Nutr..

[18] Grosso G., Galvano F. (2016). Mediterranean diet adherence in children and adolescents in southern European countries. NFS J..

[19] Cavallo M., Morgana G., Dozzani I., Gatti A., Vandoni M., Pippi R., Pucci G., Vaudo G., Fanelli C.G. (2023). Unraveling Barriers to a Healthy Lifestyle: Understanding Barriers to Diet and Physical Activity in Patients with Chronic Non-Communicable Diseases. Nutrients.

[20] Wang K., Niu Y., Lu Z., Duo B., Effah C.Y., Guan L. (2023). The effect of breakfast on childhood obesity: A systematic review and meta-analysis. Front. Nutr..

[21] Nago E.S., Lachat C.K., Dossa R.A.M., Kolsteren P.W. (2014). Association of out-of-home eating with anthropometric changes: A systematic review of prospective studies. Crit. Rev. Food Sci. Nutr..

[22] Snuggs S., Harvey K. (2023). Family mealtimes: A systematic umbrella review of characteristics, correlates, outcomes and interventions. Nutrients.

[23] Arundell L., Fletcher E., Salmon J., Veitch J., Hinkley T. (2016). A systematic review of the prevalence of sedentary behavior during the after-school period among children aged 5–18 years. Int. J. Behav. Nutr. Phys. Act..

[24] Guthold R., Stevens G.A., Riley L.M., Bull F.C. (2020). Global trends in insufficient physical activity among adolescents: A pooled analysis of 298 population-based surveys with 1.6 million participants. Lancet Child. Adolesc. Health.

[25] Biddle S.J.H., Pearson N., Ross G.M., Braithwaite R. (2010). Tracking of sedentary behaviours of young people: A systematic review. Prev. Med..

[26] Degli Innocenti P., Rosi A., Bergamo F., Scazzina F. (2024). Dietary and lifestyle intervention strategies to tackle unhealthy behaviours in the Mediterranean countries. Int. J. Food Sci. Nutr..

[27] Grosso G., Buso P., Mata A., Abdelkarim O., Aly M., Pinilla J., Fernandez A., Mendez R., Alvarez A., Valdes N. (2024). Understanding consumer food choices & promotion of healthy and sustainable Mediterranean diet and lifestyle in children and adolescents through behavioural change actions: The DELICIOUS project. Int. J. Food Sci. Nutr..

[28] Villodres G.C., Salvador Pérez F., Muros J.J. (2024). Factors associated with Mediterranean diet adherence in a sample of high socio-economic status children from southern Spain. Public. Health Nutr..

[29] Sanmarchi F., Esposito F., Marini S., Masini A., Scrimaglia S., Capodici A., Arrichiello F., Ferretti F., Rangone M., Celenza F. (2022). Children’s and Families’ Determinants of Health-Related Behaviors in an Italian Primary School Sample: The “Seven Days for My Health” Project. Int. J. Environ. Res. Public. Health.

[30] Rosi A., Biasini B., Donati M., Ricci C., Scazzina F. (2020). Adherence to the mediterranean diet and environmental impact of the diet on primary school children living in Parma (Italy). Int. J. Environ. Res. Public. Health.

[31] Rosi A., Giopp F., Milioli G., Melegari G., Goldoni M., Parrino L., Scazzina F. (2020). Weight status, adherence to the mediterranean diet, physical activity level, and sleep behavior of italian junior high school adolescents. Nutrients.

[32] Bonaccorsi G., Furlan F., Scocuzza M., Lorini C. (2020). Adherence to Mediterranean Diet among Students from Primary and Middle School in the Province of Taranto, 2016–2018. Int. J. Environ. Res. Public Health.

[33] Kuczmarski R.J., Ogden C.L., Guo S.S., Grummer-Strawn L.M., Flegal K.M., Mei Z., Wei R., Curtin L.R., Roche A.F., Johnson C.L. (2002). 2000 CDC Growth Charts for the United States: Methods and development. Vital Health Stat..

[34] Lazarou C., Panagiotakos D.B., Spanoudis G., Matalas A.-L. (2011). E-KINDEX: A dietary screening tool to assess children’s obesogenic dietary habits. J. Am. Coll. Nutr..

[35] Lee P.H., Macfarlane D.J., Lam T.H., Stewart S.M. (2011). Validity of the International Physical Activity Questionnaire Short Form (IPAQ-SF): A systematic review. Int. J. Behav. Nutr. Phys. Act..

[36] Hirshkowitz M., Whiton K., Albert S.M., Alessi C., Bruni O., DonCarlos L., Hazen N., Herman J., Adams Hillard P.J., Katz E.S. (2015). National Sleep Foundation’s updated sleep duration recommendations: Final report. Sleep. Health.

[37] Serra-Majem L., Ribas L., Ngo J., Ortega R.M., García A., Pérez-Rodrigo C., Aranceta J. (2004). Food, youth and the Mediterranean diet in Spain. Development of KIDMED, Mediterranean Diet Quality Index in children and adolescents. Public. Health Nutr..

[38] Rosi A., Scazzina F., Giampieri F., Abdelkarim O., Aly M., Pons J., Vázquez-Araújo L., Frias-Toral E., Cano S.S., Elío I. (2024). Adherence to the Mediterranean diet in 5 Mediterranean countries: A descriptive analysis of the DELICIOUS project. Med. J. Nutr. Metab..

[39] Giampieri F., Rosi A., Scazzina F., Frias-Toral E., Abdelkarim O., Aly M., Zambrano-Villacres R., Pons J., Vázquez-Araújo L., Sumalla Cano S. (2024). Youth Healthy Eating Index (YHEI) and Diet Adequacy in Relation to Country-Specific National Dietary Recommendations in Children and Adolescents in Five Mediterranean Countries from the DELICIOUS Project. Nutrients.

[40] Rutigliano I., Mansueto M.L., Canestrale R., Giorgio R., Sacco M., Pastore M.R. (2024). Children’s diet assessed with the Mediterranean Diet Index: The finding of new eating habits and their impact on a cohort of Italian children. Ann. Ist. Super. Sanita.

[41] Acito M., Valentino R., Rondini T., Fatigoni C., Moretti M., Villarini M. (2024). Mediterranean Diet Adherence in Italian Children: How much do Demographic Factors and Socio-Economic Status Matter?. Matern. Child. Health J..

[42] Calderón García A., Pedrero Tomé R., Alaminos Torres A., Prado Martínez C., Martínez Álvarez J.R., López Ejeda N., García Rodríguez M., Marrodán Serrano M.D. (2024). Adherence to the Mediterranean diet and eating behaviour in Spanish schoolchildren. Nutr. Hosp..

[43] Marques G.F.S., Pinto S.M.O., Reis A.C.R.d.S., Martins T.D.B., Conceição A.P.d., Pinheiro A.R.V. (2021). Adherence to the mediterranean diet in elementary school children (1st cycle). Rev. Paul. Pediatr..

[44] Mignogna C., Costanzo S., Ghulam A., Cerletti C., Donati M.B., de Gaetano G., Iacoviello L., Bonaccio M. (2022). Impact of Nationwide Lockdowns Resulting from the First Wave of the COVID-19 Pandemic on Food Intake, Eating Behaviors, and Diet Quality: A Systematic Review. Adv. Nutr..

[45] Galan-Lopez P., Sánchez-Oliver A.J., Ries F., González-Jurado J.A. (2019). Mediterranean diet, physical fitness and body composition in sevillian adolescents: A healthy lifestyle. Nutrients.

[46] Santomauro F., Lorini C., Tanini T., Indiani L., Lastrucci V., Comodo N., Bonaccorsi G. (2014). Adherence to Mediterranean diet in a sample of Tuscan adolescents. Nutrition.

[47] del Mar Bibiloni M., Martínez E., Llull R., Pons A., Tur J.A. (2012). Western and Mediterranean dietary patterns among Balearic Islands’ adolescents: Socio-economic and lifestyle determinants. Public. Health Nutr..

[48] Kontogianni M.D., Vidra N., Farmaki A.-E., Koinaki S., Belogianni K., Sofrona S., Magkanari F., Yannakoulia M. (2008). Adherence rates to the Mediterranean diet are low in a representative sample of Greek children and adolescents. J. Nutr..

[49] Bôto J.M., Marreiros A., Diogo P., Pinto E., Mateus M.P. (2022). Health behaviours as predictors of the Mediterranean diet adherence: A decision tree approach. Public. Health Nutr..

[50] Giménez-Legarre N., Santaliestra-Pasías A.M., De Henauw S., Forsner M., González-Gross M., Jurado-Fasoli L., Kafatos A., Karaglani E., Lambrinou C.-P., Molnár D. (2022). Breakfast consumption and its relationship with diet quality and adherence to Mediterranean diet in European adolescents: The HELENA study. Eur. J. Clin. Nutr..

[51] Mounayar R., Jreij R., Hachem J., Abboud F., Tueni M. (2019). Breakfast Intake and Factors Associated with Adherence to the Mediterranean Diet among Lebanese High School Adolescents. J. Nutr. Metab..

[52] Pearson N., Biddle S.J.H., Gorely T. (2009). Family correlates of breakfast consumption among children and adolescents. A systematic review. Appetite.

[53] Abdelkarim O., El-Gyar N., Shalaby A.M., Aly M. (2024). The Effects of a School-Based Physical Activity Program on Physical Fitness in Egyptian Children: A Pilot Study from the DELICIOUS Project. Children.

[54] Hills A.P., Andersen L.B., Byrne N.M. (2011). Physical activity and obesity in children. Br. J. Sports Med..

[55] Carson V., Hunter S., Kuzik N., Gray C.E., Poitras V.J., Chaput J.-P., Saunders T.J., Katzmarzyk P.T., Okely A.D., Connor Gorber S. (2016). Systematic review of sedentary behaviour and health indicators in school-aged children and youth: An update. Appl. Physiol. Nutr. Metab..

[56] García-Hermoso A., Ezzatvar Y., López-Gil J.F., Ramírez-Vélez R., Olloquequi J., Izquierdo M. (2022). Is adherence to the Mediterranean diet associated with healthy habits and physical fitness? A systematic review and meta-analysis including 565,421 youths. Br. J. Nutr..

[57] Mitri R.N., Boulos C., Ziade F. (2022). Mediterranean diet adherence amongst adolescents in North Lebanon: The role of skipping meals, meals with the family, physical activity and physical well-being. Br. J. Nutr..

[58] Sanz-Martín D., Zurita-Ortega F., Puertas-Molero P., Caracuel-Cáliz R., Alonso-Vargas J.M., Melguizo-Ibáñez E. (2023). Relationship between Physical Activity, Mediterranean Diet and Emotional Intelligence in Spanish Primary Education Students. Children.

[59] Pérez-Mármol M., Chacón-Cuberos R., García-Mármol E., Castro-Sánchez M. (2021). Relationships among Physical Self-Concept, Physical Activity and Mediterranean Diet in Adolescents from the Province of Granada. Children.

[60] Melguizo-Ibáñez E., González-Valero G., Puertas-Molero P., Alonso-Vargas J.M. (2022). Emotional Intelligence, Physical Activity Practice and Mediterranean Diet Adherence-An Explanatory Model in Elementary Education School Students. Children.

[61] Martino F., Puddu P.E., Lamacchia F., Colantoni C., Zanoni C., Barillà F., Martino E., Angelico F. (2016). Mediterranean diet and physical activity impact on metabolic syndrome among children and adolescents from Southern Italy: Contribution from the Calabrian Sierras Community Study (CSCS). Int. J. Cardiol..

[62] Godos J., Ferri R., Lanza G., Caraci F., Vistorte A.O.R., Yelamos Torres V., Grosso G., Castellano S. (2024). Mediterranean diet and sleep features: A systematic review of current evidence. Nutrients.

[63] Cespedes E.M., Hu F.B., Redline S., Rosner B., Gillman M.W., Rifas-Shiman S.L., Taveras E.M. (2016). Chronic insufficient sleep and diet quality: Contributors to childhood obesity. Obesity.

[64] Godos J., Lanza G., Ferri R., Caraci F., Cano S.S., Elio I., Micek A., Castellano S., Grosso G. (2024). Relation between dietary inflammatory potential and sleep features: Systematic review of observational studies. Med. J. Nutr. Metab..

[65] Grosso G., Marventano S., Buscemi S., Scuderi A., Matalone M., Platania A., Giorgianni G., Rametta S., Nolfo F., Galvano F. (2013). Factors associated with adherence to the Mediterranean diet among adolescents living in Sicily, Southern Italy. Nutrients.

[66] Seral-Cortes M., Sabroso-Lasa S., Bailo-Aysa A., Gonzalez-Gross M., Molnár D., Censi L., Molina-Hidalgo C., Gottrand F., Henauw S.D., Manios Y. (2021). Mediterranean Diet, Screen-Time-Based Sedentary Behavior and Their Interaction Effect on Adiposity in European Adolescents: The HELENA Study. Nutrients.

[67] Bawaked R.A., Gomez S.F., Homs C., Casas Esteve R., Cardenas G., Fíto M., Schröder H. (2018). Association of eating behaviors, lifestyle, and maternal education with adherence to the Mediterranean diet in Spanish children. Appetite.

[68] Wärnberg J., Pérez-Farinós N., Benavente-Marín J.C., Gómez S.F., Labayen I., Zapico A.G., Gusi N., Aznar S., Alcaraz P.E., González-Valeiro M. (2021). Screen Time and Parents’ Education Level Are Associated with Poor Adherence to the Mediterranean Diet in Spanish Children and Adolescents: The PASOS Study. J. Clin. Med..

[69] Zerón-Rugerio M.F., Santamaría-Orleans A., Izquierdo-Pulido M. (2024). Late bedtime combined with more screen time before bed increases the risk of obesity and lowers diet quality in Spanish children. Appetite.

[70] López-Gil J.F., Brazo-Sayavera J., de Campos W., Yuste Lucas J.L. (2020). Meeting the Physical Activity Recommendations and Its Relationship with Obesity-Related Parameters, Physical Fitness, Screen Time, and Mediterranean Diet in Schoolchildren. Children.

[71] Rosi A., Calestani M.V., Parrino L., Milioli G., Palla L., Volta E., Brighenti F., Scazzina F. (2017). Weight Status Is Related with Gender and Sleep Duration but Not with Dietary Habits and Physical Activity in Primary School Italian Children. Nutrients.

[72] Álvarez C., Guzmán-Guzmán I.P., Latorre-Román P.Á., Párraga-Montilla J., Palomino-Devia C., Reyes-Oyola F.A., Paredes-Arévalo L., Leal-Oyarzún M., Obando-Calderón I., Cresp-Barria M. (2021). Association between the Sociodemographic Characteristics of Parents with Health-Related and Lifestyle Markers of Children in Three Different Spanish-Speaking Countries: An Inter-Continental Study at OECD Country Level. Nutrients.

[73] El Mokhtari O., Anzid K., Levy-Desroches S., del Montero López M.P., Cherkaoui M., Hilali A. (2024). Adherence to the Mediterranean Diet among high-school pupils in the North Moroccan Rif region. Med. J. Nutr. Metab..

[74] Munt A.E., Partridge S.R., Allman-Farinelli M. (2017). The barriers and enablers of healthy eating among young adults: A missing piece of the obesity puzzle: A scoping review. Obes. Rev..

[75] Grady A., Jackson J.K., Lum M., Delaney T., Jones J., Kerr J., Falkiner M., Yoong S. (2022). Barriers and facilitators to the implementation of healthy eating, physical activity and obesity prevention policies, practices or programs in family day care: A mixed method systematic review. Prev. Med..

[76] Scaglioni S., De Cosmi V., Ciappolino V., Parazzini F., Brambilla P., Agostoni C. (2018). Factors influencing children’s eating behaviours. Nutrients.

[77] Pereira-da-Silva L., Rêgo C., Pietrobelli A. (2016). The diet of preschool children in the mediterranean countries of the european union: A systematic review. Int. J. Environ. Res. Public. Health.

[78] Grassi T., Bagordo F., Panico A., De Giorgi M., Idolo A., Serio F., Tumolo M.R., De Donno A. (2020). Adherence to Mediterranean diet of children living in small Southern Italian villages. Int. J. Food Sci. Nutr..

[79] Buja A., Grotto G., Brocadello F., Sperotto M., Baldo V. (2020). Primary school children and nutrition: Lifestyles and behavioral traits associated with a poor-to-moderate adherence to the Mediterranean diet. A cross-sectional study. Eur. J. Pediatr..

[80] Bibiloni M.D.M., Gallardo-Alfaro L., Gómez S.F., Wärnberg J., Osés-Recalde M., González-Gross M., Gusi N., Aznar S., Marín-Cascales E., González-Valeiro M.A. (2022). Determinants of adherence to the mediterranean diet in spanish children and adolescents: The PASOS study. Nutrients.

[81] Herrera-Ramos E., Tomaino L., Sánchez-Villegas A., Ribas-Barba L., Gómez S.F., Wärnberg J., Osés M., González-Gross M., Gusi N., Aznar S. (2023). Trends in Adherence to the Mediterranean Diet in Spanish Children and Adolescents across Two Decades. Nutrients.

[82] Iaccarino Idelson P., Scalfi L., Valerio G. (2017). Adherence to the Mediterranean Diet in children and adolescents: A systematic review. Nutr. Metab. Cardiovasc. Dis..

[83] Bonaccio M., Di Castelnuovo A., Costanzo S., De Lucia F., Olivieri M., Donati M.B., de Gaetano G., Iacoviello L., Bonanni A. (2013). Moli-sani Project Investigators Nutrition knowledge is associated with higher adherence to Mediterranean diet and lower prevalence of obesity. Results from the Moli-sani study. Appetite.

[84] Aureli V., Rossi L. (2022). Nutrition knowledge as a driver of adherence to the mediterranean diet in italy. Front. Nutr..

[85] Yassıbaş E., Bölükbaşı H. (2023). Evaluation of adherence to the Mediterranean diet with sustainable nutrition knowledge and environmentally responsible food choices. Front. Nutr..

