# Supplementary material for: Lifestyle Factors Associated with Children’s and Adolescents’ Adherence to the Mediterranean Diet Living in Mediterranean Countries: The DELICIOUS Project
Source: Nutrients. 2024 Dec 25;17(1):26. doi: 10.3390/nu17010026 (PMC11722675; doi:10.3390/nu17010026)
Supplement: Supplementary file 1 [file nutrients-17-00026-s001.zip › nutrients-3345510-supplementary.pdf]

Supplementary Table S1. Factors associated with high adherence to the Mediterranean diet in individual countries participating in the study.

	**Lebanon**	**Italy**	**Spain**	**Egypt**	**Portugal**
	*OR (95% CI) ***				
Parents age					
<44 y	1	1	1	1	1
≥45 y	0.62 (0.38, 1.02)	0.62 (0.28, 1.38)	0.88 (0.37, 2.08)	0.48 (0.31, 0.76)	1.37 (0.52, 3.59)
Parents educational level					
Low	1	1	1	1	1
Medium	4.05 (0.87, 18.90)	1.20 (0.52, 2.78)	1.87 (0.67, 5.21)	2.32 (0.31, 17.41)	1.98 (0.21, 18.81)
High	8.16 (1.82, 36.62)	1.46 (0.63, 3.41)	2.53 (0.89, 7.23)	1.43 (0.24, 8.58)	3.05 (0.32, 29.12)
Age groups					
6-8 y	1	1	1	1	1
9-11 y	1.04 (0.58, 1.89)	1.08 (0.58, 2.02)	0.93 (0.52, 1.67)	1.09 (0.59, 2.02)	0.96 (0.54, 1.72)
12-14 y	1.29 (0.68, 2.45)	0.87 (0.46, 1.66)	1.09 (0.60, 1.98)	1.04 (0.55, 1.96)	0.44 (0.24, 0.81)
15-17 y	0.53 (0.27, 1.05)	0.91 (0.48, 1.73)	0.75 (0.41, 1.35)	1.21 (0.65, 2.25)	0.38 (0.20, 0.72)
Physical activity level					
Low	1	1	1	1	1
Medium	1.26 (0.67, 2.40)	2.40 (1.39, 4.19)	2.47 (1.43, 4.26)	2.57 (1.54, 4.26)	1.55 (0.91, 2.64)
High	1.23 (0.72, 2.09)	2.17 (1.29, 3.65)	1.98 (1.23, 3.19)	2.88 (1.60, 5.22)	1.37 (0.83, 2.27)
Sleep duration					
Less than 8 hrs	1	1	1	1	1
8-10 hrs	1.53 (0.90, 2.60)	1.35 (0.76, 2.41)	1.49 (0.74, 2.99)	1.92 (1.08, 3.39)	0.78 (0.40, 1.49)
>10 hrs	1.55 (0.51, 4.69)	1.59 (0.54, 4.68)	2.05 (0.64, 6.60)	0.97 (0.36, 2.59)	0.45 (0.04, 5.16)
Healthy lifestyle score*					
Low	1	1	1	1	1
Medium	1.31 (0.79, 2.17)	1.24 (0.67, 2.30)	0.74 (0.33, 1.66)	1.91 (1.16, 3.14)	1.29 (0.75, 2.21)
High	0.80 (0.30, 2.10)	1.57 (0.88, 2.80)	1.41 (0.70, 2.84)	0.76 (0.21, 2.83)	1.53 (0.92, 2.56)

*based on the E-KINDEX score

**Analyses were adjusted for all variables presented in the table.
